# Supplementary material for: ENDOG Impacts on Tumor Cell Proliferation and Tumor Prognosis in the Context of PI3K/PTEN Pathway Status
Source: Cancers (Basel). 2021 Jul 28;13(15):3803. doi: 10.3390/cancers13153803 (PMC8345062; doi:10.3390/cancers13153803)

Figure 1C

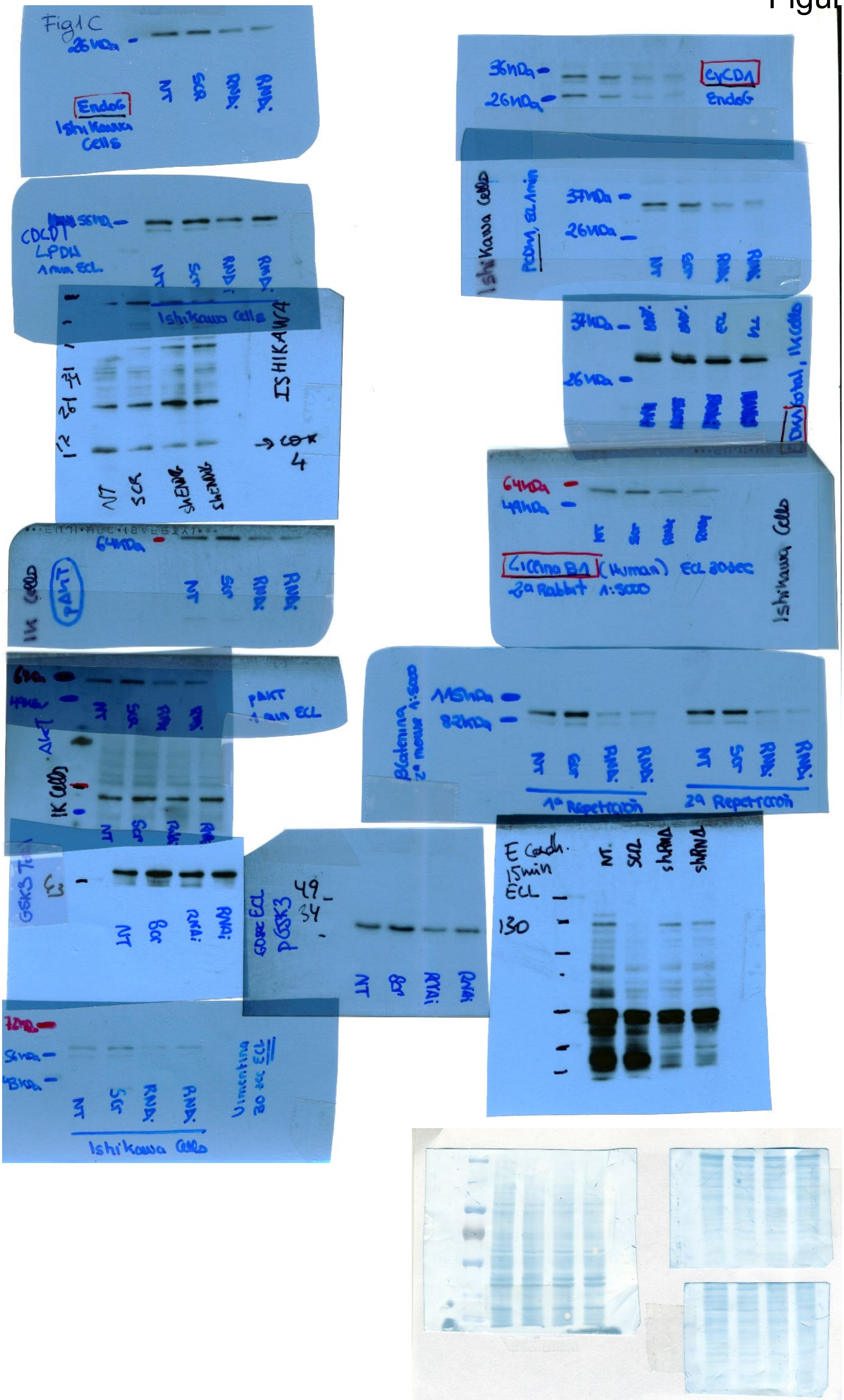

Fig1C
EndoG
Ishikawa Cells
NT
SCR
CyCD1
EndoG
CDCD1
LPDH
1min ECL
Ishikawa Cells
ISHIKAWA
NT
SCR
shENDG
pAKT
1min ECL
AKT
IK Cells
GSK3
PGSK3
Vimentina
30 sec ECL
Ishikawa Cells
Ishikawa Cells
Ciclina B1 (Human) ECL 20sec
2a Rabbit 1:5000
Ishikawa Cells
B-catenina
2a mouse 1:5000
1a Repeticion
2a Repeticion
E Cadh.
15min
ECL
130
shRNA

Figure 2A

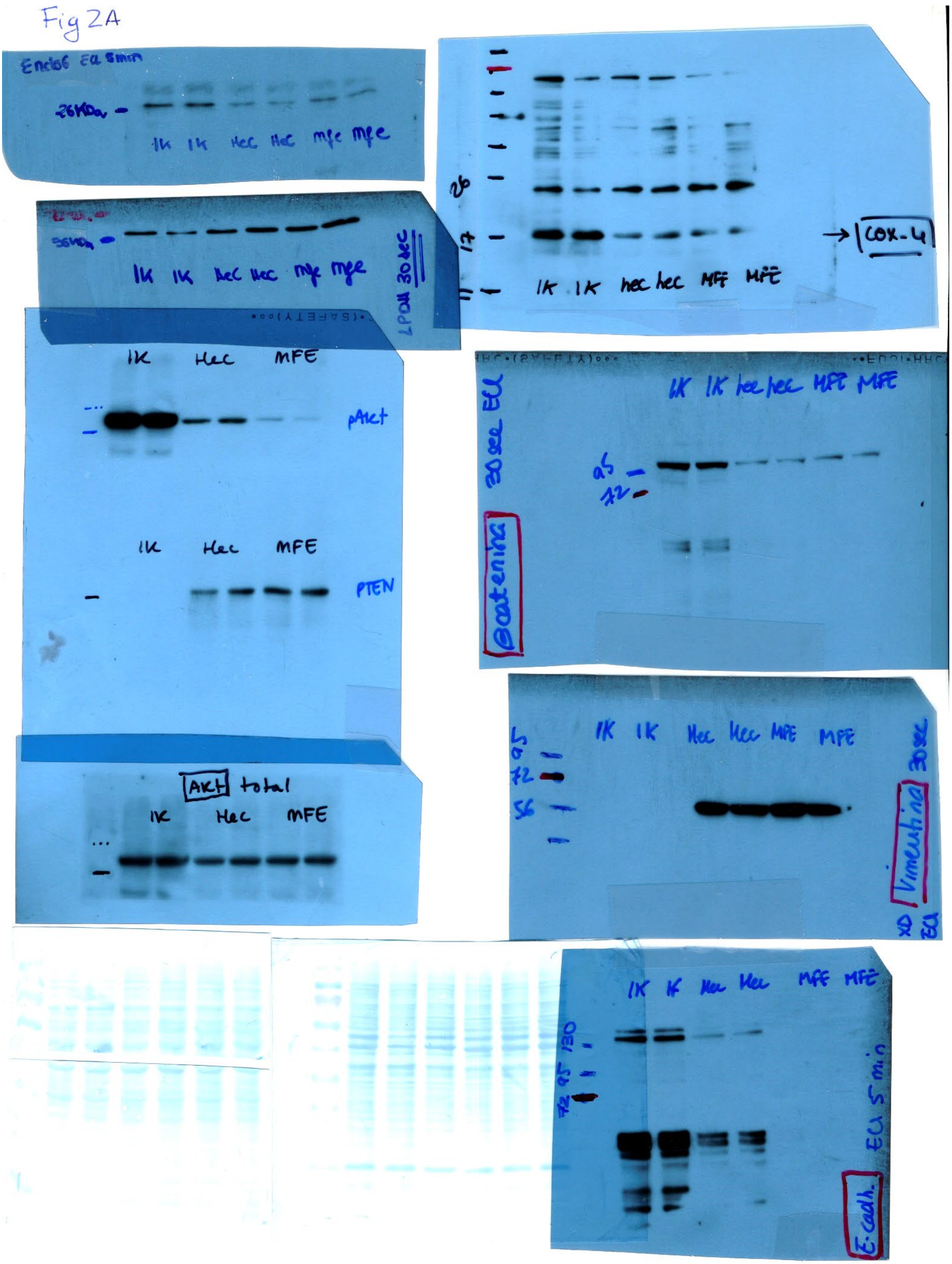
Fig 2A
26KDa
56KDa
COX-4
pAkt
PTEN
AKt total
βcatenina
30 sec ECL
95
72
56
Vimentina
30 sec
130
E.cadh.
ECL 5 min

Figure 3C

Fig 3A.

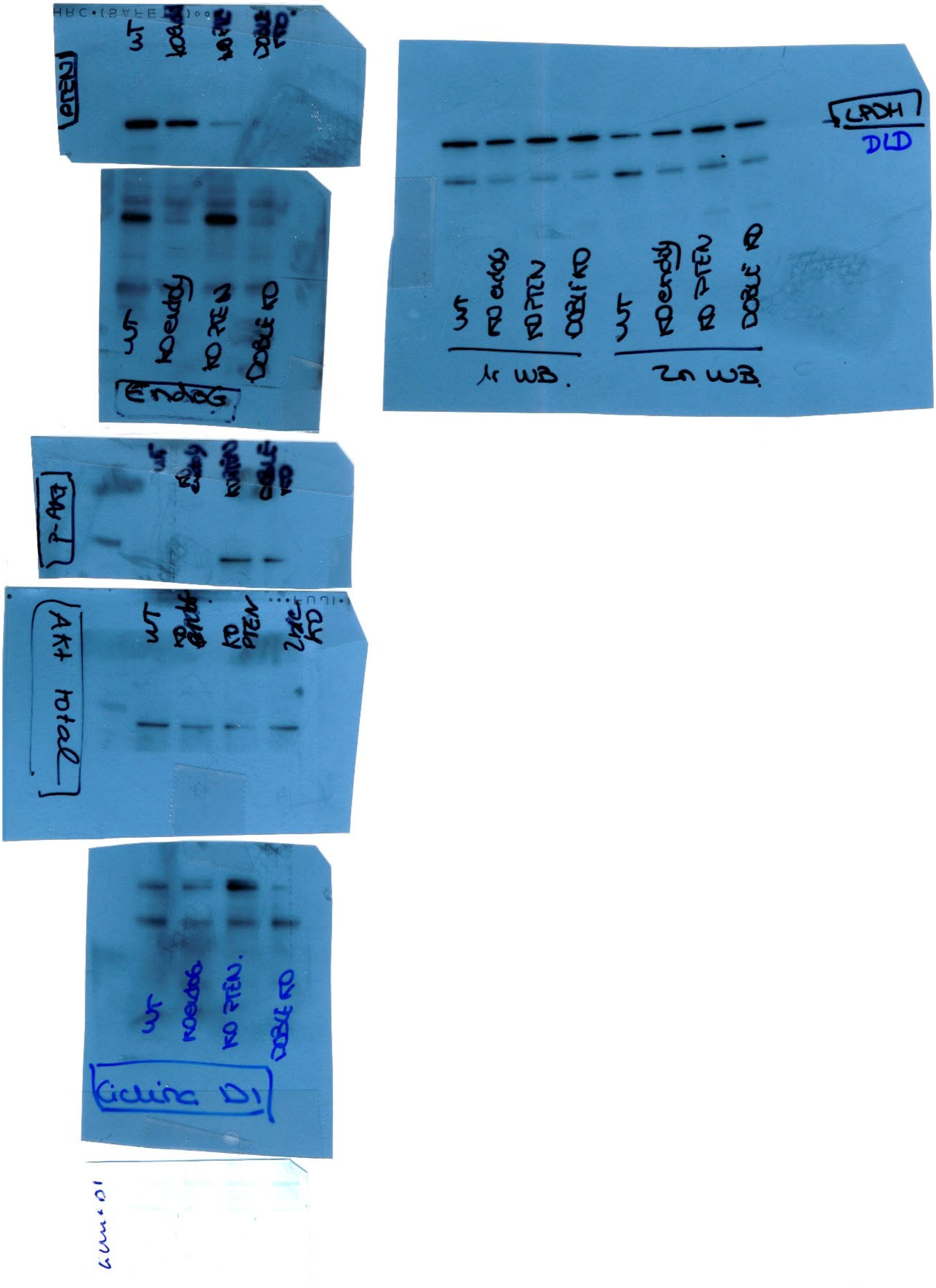

Figure 4B

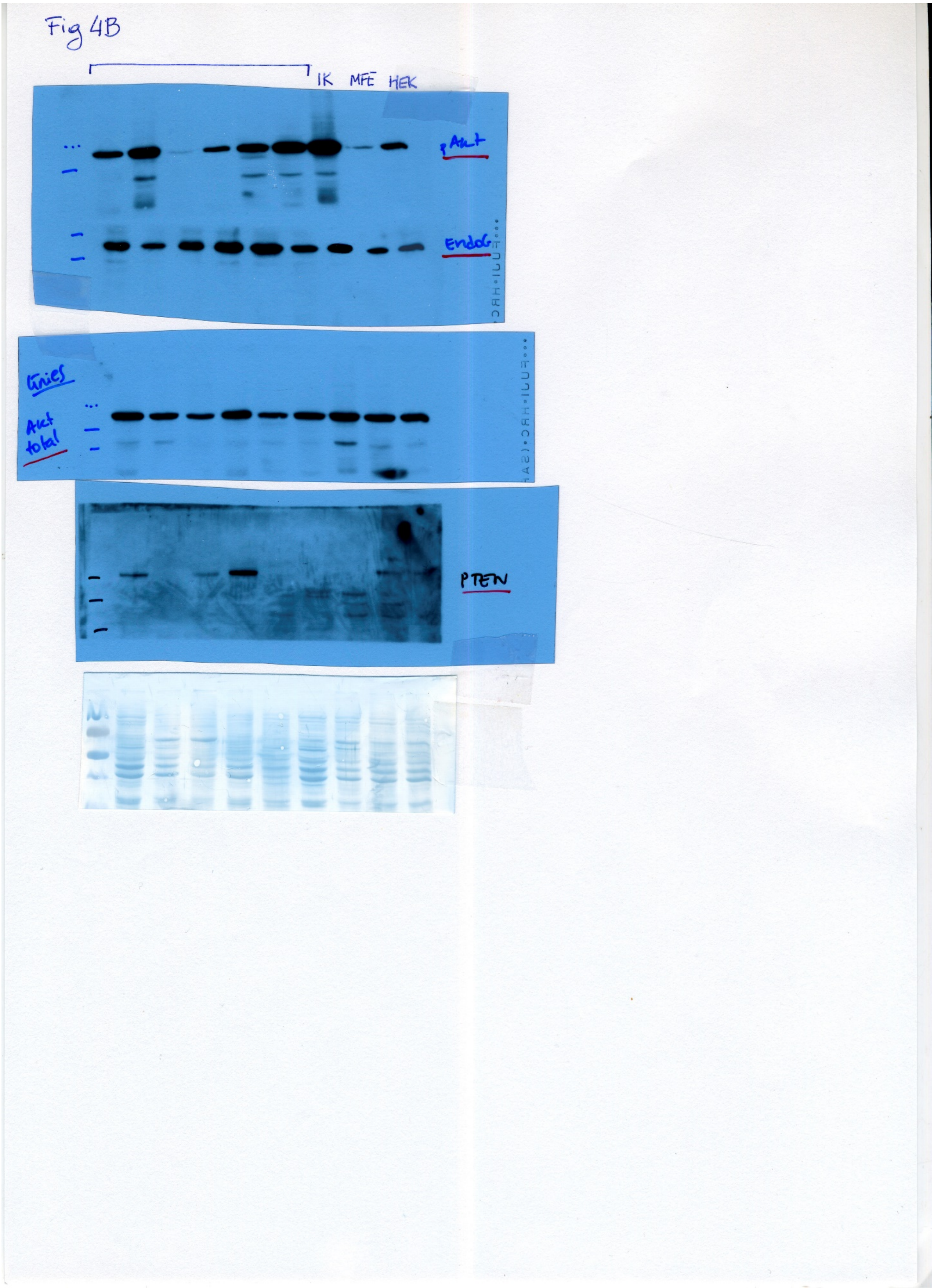

Supplement: Supplementary file 1 [file cancers-13-03803-s001.zip › cancers-1287394-suppl-xml/cancers-1180439-origianl western blot.pdf]
