# Supplementary material for: ENDOG Impacts on Tumor Cell Proliferation and Tumor Prognosis in the Context of PI3K/PTEN Pathway Status
Source: Cancers (Basel). 2021 Jul 28;13(15):3803. doi: 10.3390/cancers13153803 (PMC8345062; doi:10.3390/cancers13153803)
Supplement: Supplementary file 1 [file cancers-13-03803-s001.zip › cancers-1287394-suppl-xml/cancers-1287394-supplementary figures.pdf]

Supplementary Materials

ENDOG Impacts on Tumor Cell Proliferation and Tumor Prognosis in the Context of PI3K/PTEN Pathway Status

Gisel Barés, Aida Beà, Luís Hernández, Raul Navaridas, Isidre Felip, Cristina Megino, Natividad Blasco, Ferran Nadeu, Elías Campo, Marta Llovera, Xavier Dolcet and Daniel Sanchis

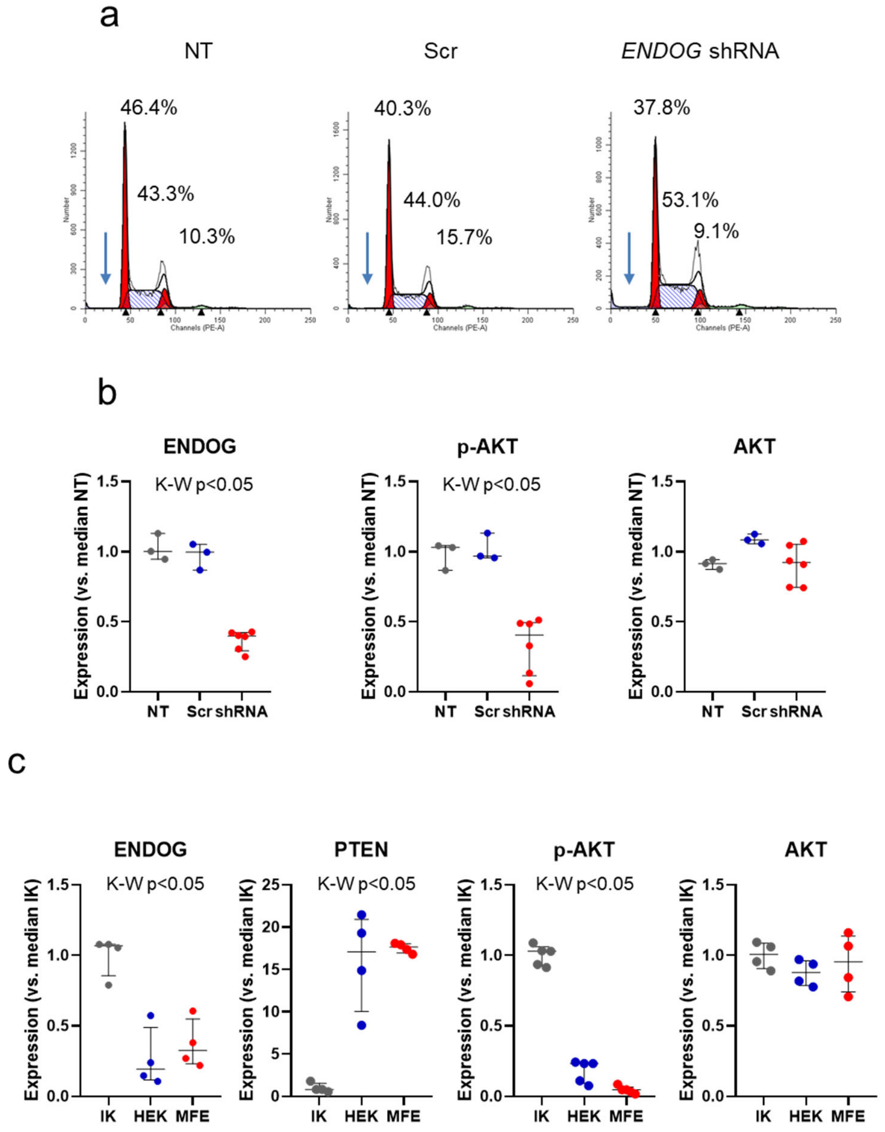

Figure S1. (**a**) Representative flow cytometry graphs obtained during experiment shown in Figure 1c. Notice the lack of sub G1 peak in Ishikawa cells with all the treatments: NT: not transduced; Scr: scrambled-transduced; *ENDOG* shRNA: *ENDOG*-specific shRNA transduced cultures. The arrow indicates the region in which dead cells appear. (**b**) Densitometric analysis of ENDOG, p-Ser473-AKT and AKT blots of IK cells in relation to Figure 1d. Kruskal-Wallis test followed by Dunn's test was performed. ENDOG and p-AKT were downregulated in shRNA ($p < 0.05$). (**c**) Densitometric analysis of ENDOG, PTEN, p-AKT and AKT blots of IK, HEK-1A and MFE-296 in relation to Figure 2a. Kruskal-Wallis test followed by Dunn's test was performed. $p < 0.05$ vs. IK protein cell lysates.

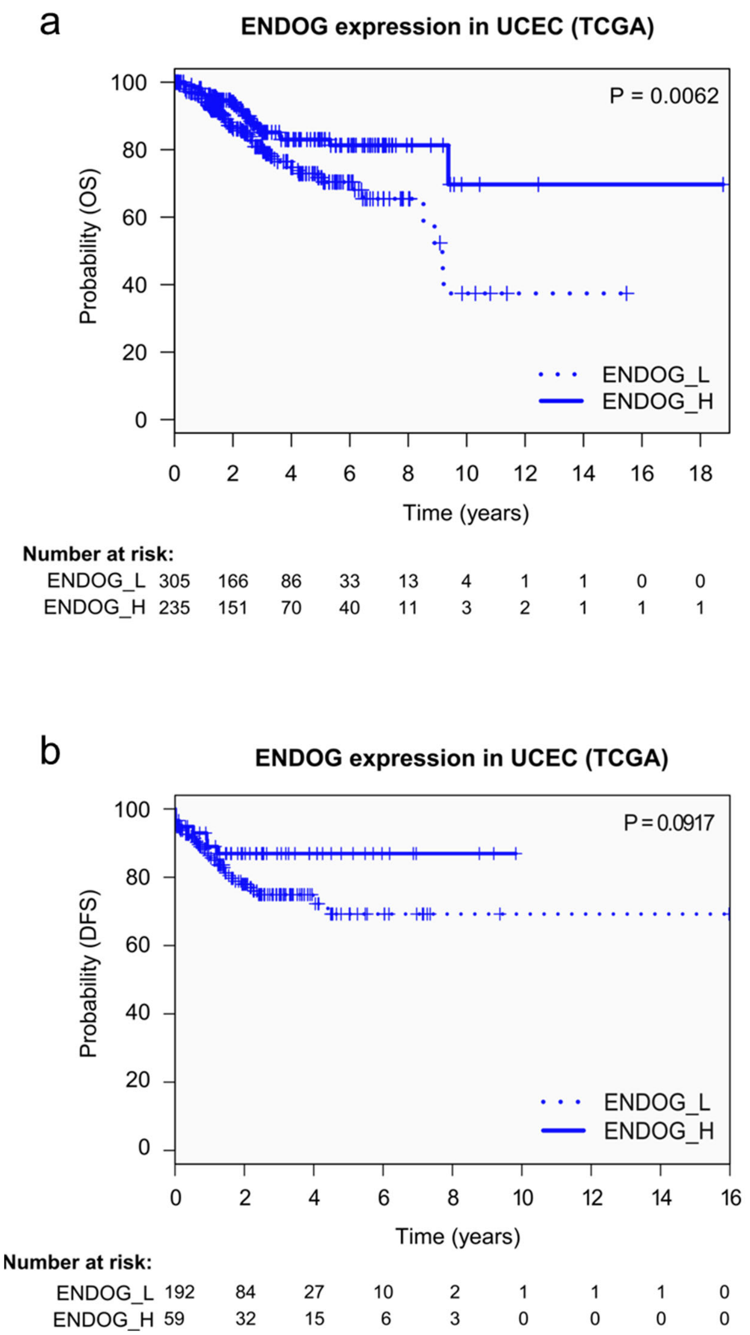

Figure S2. (**a**) Overall survival (OS) cumulative curve for patients with endometrial cancer (UCEC; TCGA project), according to *ENDOG* expression level groups (H: high expression; L: low expression)**.** UCEC patients with high (H) *ENDOG* expression levels showed a significantly longer OS compared to those with low (L) *ENDOG* expression levels. (**b**) Disease-free survival (DFS) cumulative curve for patients with endometrial cancer (UCEC; TCGA project), according to *ENDOG* expression level groups. No significant differences in DFS were detected.

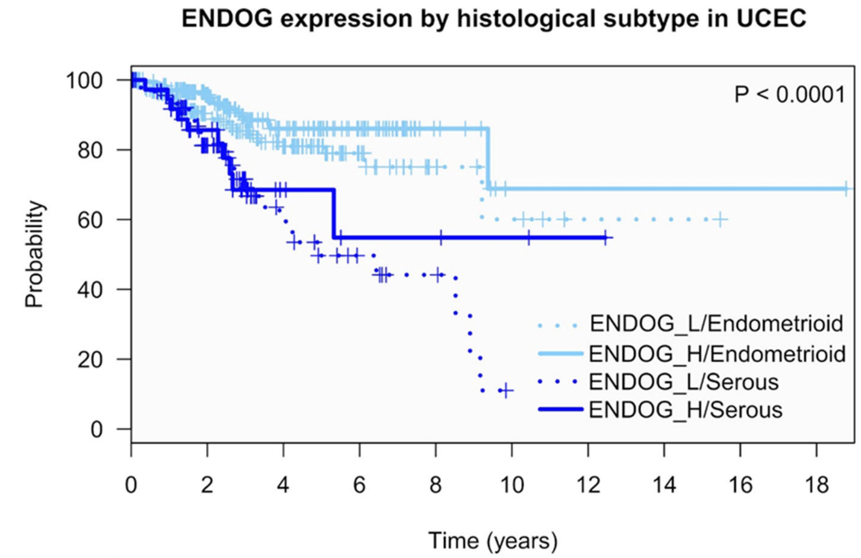

Number at risk:

ENDOG_L/Endometrioid	200	112	66	23	8	4	1	1	0	0
ENDOG_H/Endometrioid	197	126	64	37	8	1	1	1	1	1
ENDOG_L/Serous	96	51	18	9	5	0	0	0	0	0
ENDOG_H/Serous	37	24	6	3	3	2	1	0	0	0

Figure S3. Overall survival (OS) cumulative curve for patients with endometrial cancer (UCEC; TCGA project), according to *ENDOG* expression level groups (H: high expression; L: low expression) in the two major histological subtypes**.** No significant differences in OS were detected related to *ENDOG* expression levels in any of the two UCEC subtypes, although significant prognostic differences were observed only according to the subtype, being endometrioid cancers less aggressive than serous types.

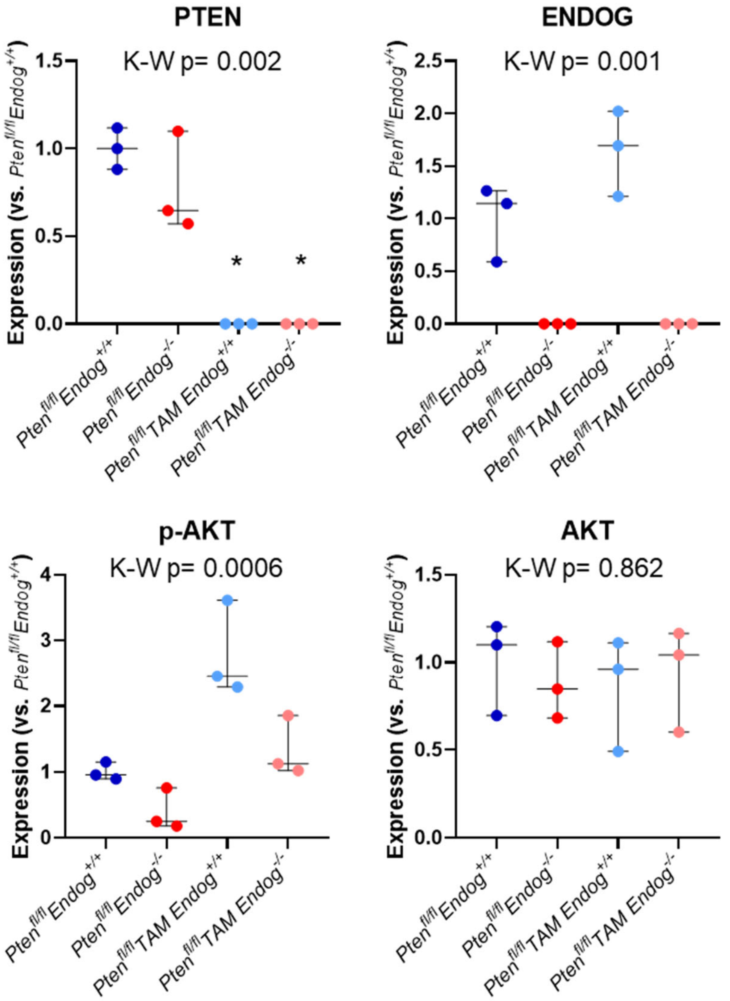

Figure S4. Densitometric analysis of ENDOG, PTEN, p-AKT and AKT blots from endometrial epithelial cell 3D cultures, N = 3 independent experiments in relation to Figure 3c. Kruskal-Wallis test (exact p value is given), followed by Dunnett's test was performed. *, $p < 0.05$.

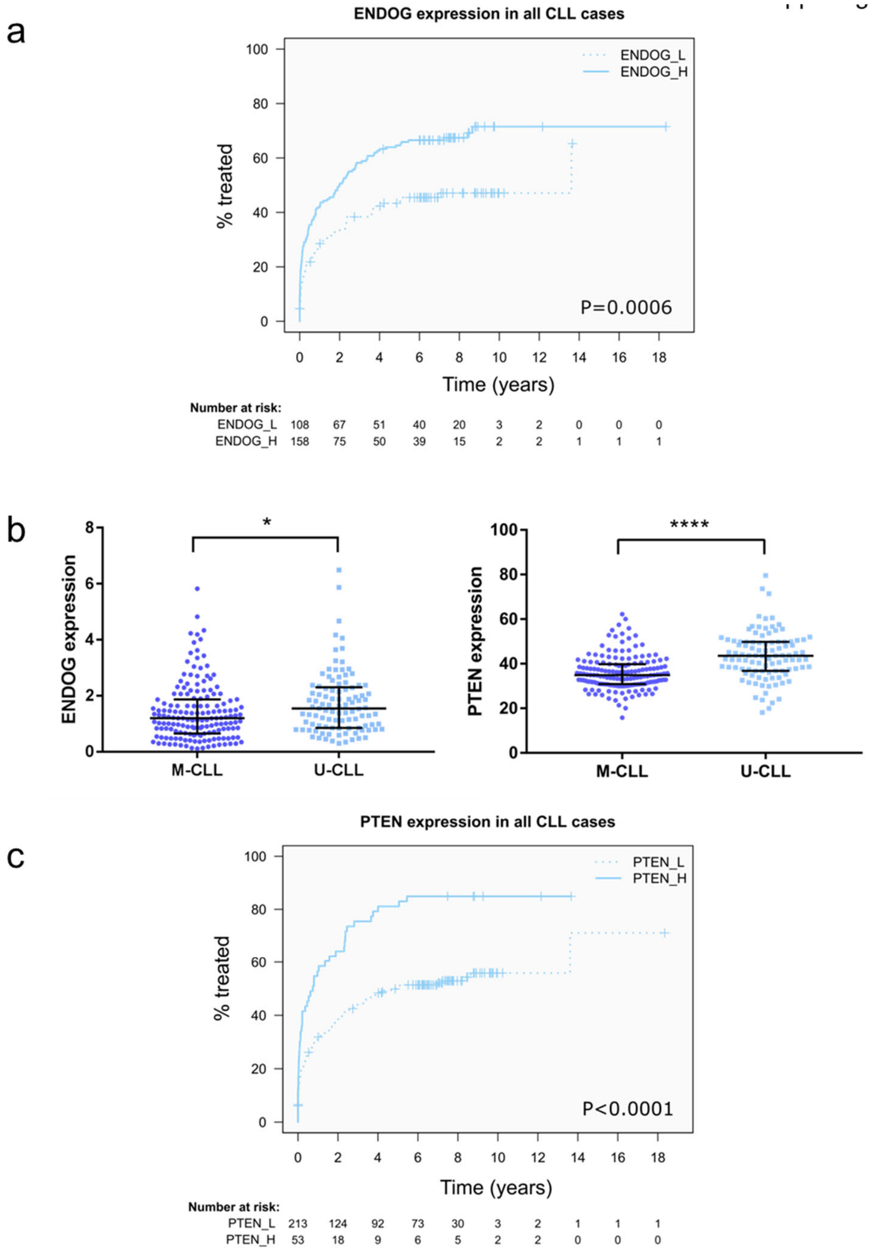

Figure S5. (**a**) Time to first treatment (TTT) cumulative curves according to *ENDOG* expression groups (H: high expression; L: low expression) in the whole CLL series. (**b**) Dot plot graphs show all individual measurements of *ENDOG* (left panel) and *PTEN* (right panel) expression among CLL subtypes defined according by their IGHV mutational status. Bars show median ± interquartile range. T-tests were performed between the subgroups. * $p < 0.05$; **** $p < 0.0001$. (**c**) TTT cumulative curves according to *PTEN* expression groups (H: high expression; L: low expression) in the whole CLL series. Maxstat algorithm was used to define the *ENDOG* and *PTEN* expression groups from the whole CLL series.

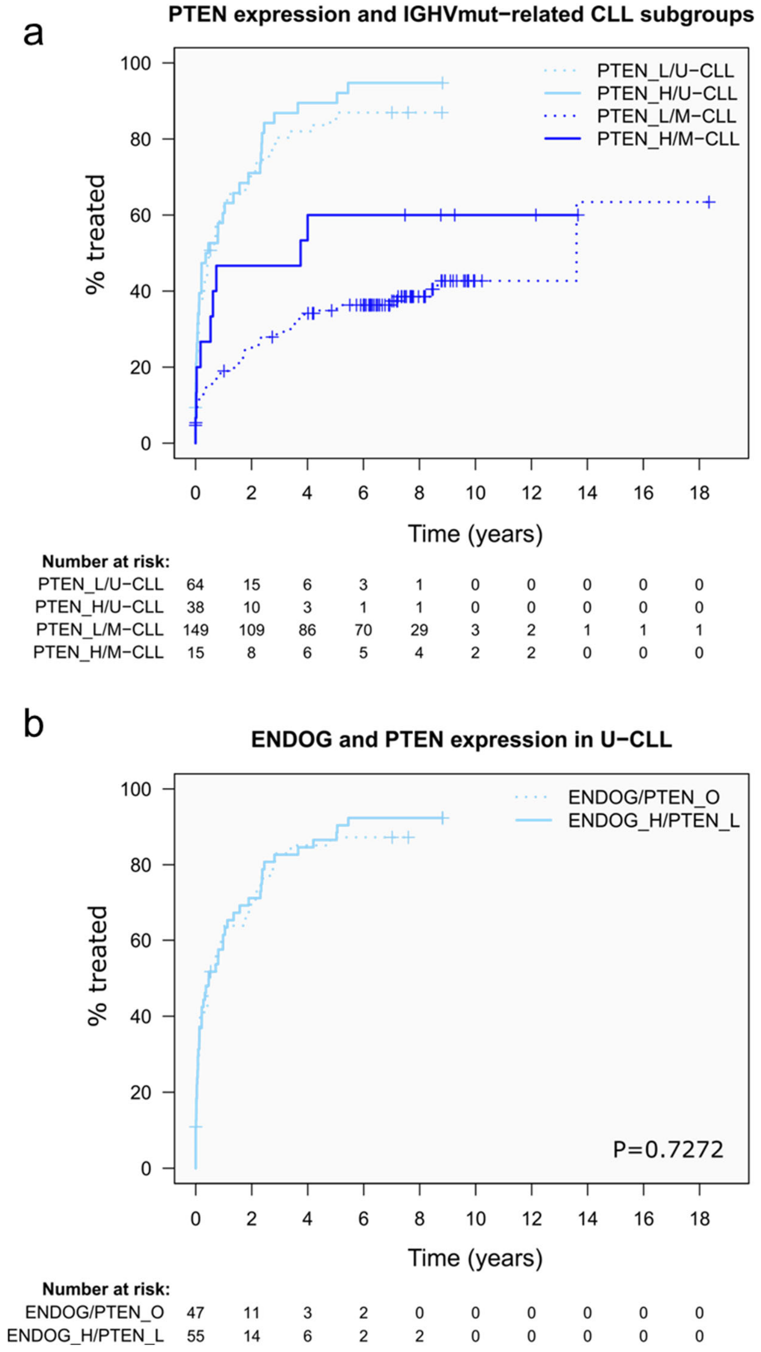

Figure S6. (**a**) TTT cumulative curves according to *PTEN* expression level groups (H: high expression; L: low expression), in subgroups of patients defined by their IGHV mutational status. No significant differences in TTT were detected according the *PTEN* expression levels found in any of the two CLL subgroups. (**b**) TTT cumulative curves according to *ENDOG* and *PTEN* expression groups in U-CLL subtype. No significant difference was detected in TTT between cases with high (H) *ENDOG*/low (L) *PTEN* levels and the remaining combinations of other (O) *ENDOG*/*PTEN* expression groups.

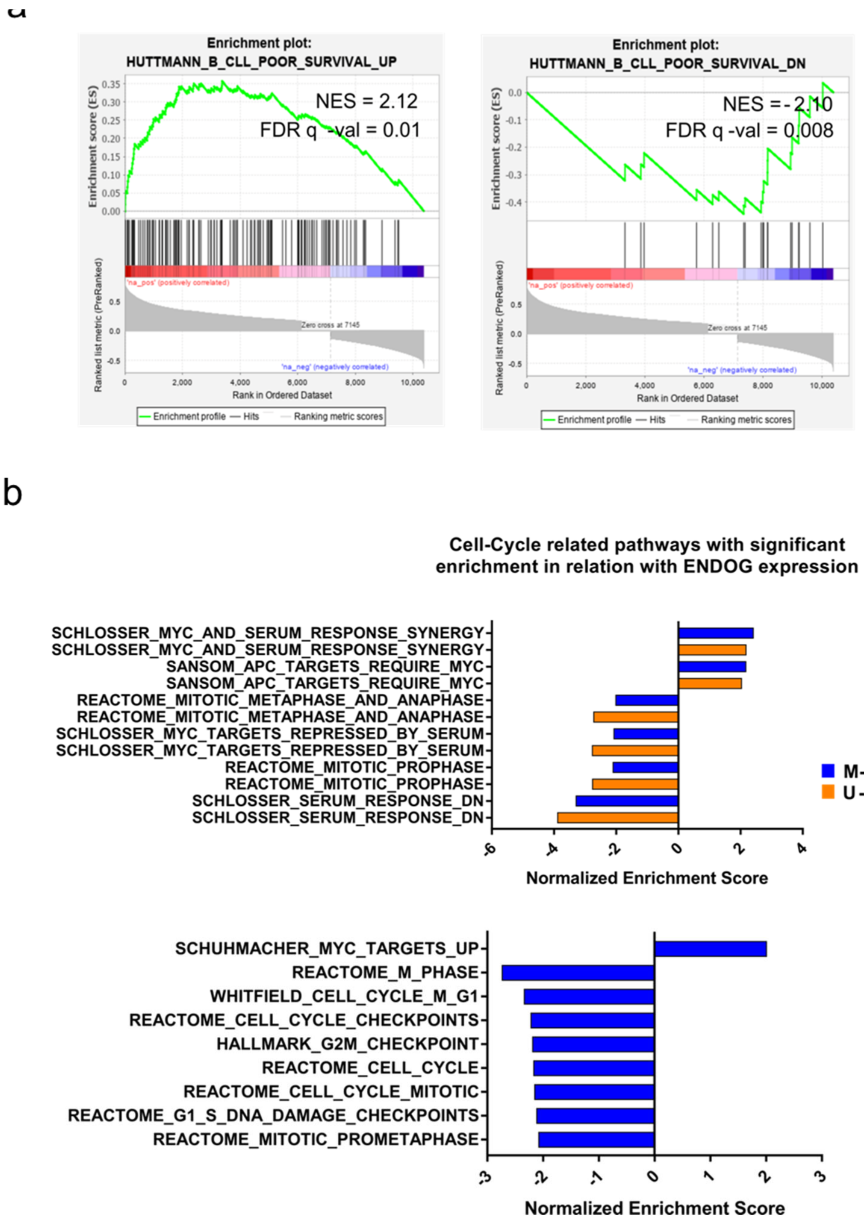

Figure S7. (**a**) Significant enrichment in signatures previously related to CLL poor prognosis were identified by GSEA either regarding positive (left panel) as negative (right panel) correlations to *ENDOG*. These signature were only found in M-CLL patients. (**b**) Summary of cell-cycle related pathways with significant enrichment identified by GSEA, including cell-cycle regulatory genes either with positive as negative correlations to *ENDOG*. Several of such pathways were found in common for M-CLL and U-CLL cases (top panel) but additional pathways were found exclusively in the M-CLL subtype (bottom panel).
